# Protein Design Using Continuous Rotamers

**DOI:** 10.1371/journal.pcbi.1002335

**Published:** 2012-01-12

**Authors:** Pablo Gainza, Kyle E. Roberts, Bruce R. Donald

**Affiliations:** 1 Department of Computer Science, Duke University, Durham, North Carolina, United States of America; 2 Department of Biochemistry, Duke University Medical Center, Durham, North Carolina, United States of America; MRC Laboratory of Molecular Biology, United Kingdom

## Abstract

Optimizing amino acid conformation and identity is a central problem in computational protein design. Protein design algorithms must allow realistic protein flexibility to occur during this optimization, or they may fail to find the best sequence with the lowest energy. Most design algorithms implement side-chain flexibility by allowing the side chains to move between a small set of discrete, low-energy states, which we call *rigid rotamers*. In this work we show that allowing continuous side-chain flexibility (which we call *continuous rotamers*) greatly improves protein flexibility modeling. We present a large-scale study that compares the sequences and best energy conformations in 69 protein-core redesigns using a rigid-rotamer model versus a continuous-rotamer model. We show that in nearly all of our redesigns the sequence found by the continuous-rotamer model is different and has a lower energy than the one found by the rigid-rotamer model. Moreover, the sequences found by the continuous-rotamer model are more similar to the native sequences. We then show that the seemingly easy solution of sampling more rigid rotamers within the continuous region is not a practical alternative to a continuous-rotamer model: at computationally feasible resolutions, using more rigid rotamers was never better than a continuous-rotamer model and almost always resulted in higher energies. Finally, we present a new protein design algorithm based on the dead-end elimination (DEE) algorithm, which we call iMinDEE, that makes the use of continuous rotamers feasible in larger systems. iMinDEE *guarantees* finding the optimal answer while pruning the search space with close to the same efficiency of DEE. **Availability:** Software is available under the Lesser GNU Public License v3. Contact the authors for source code.

## Author Summary

Computational protein design is a promising field with many biomedical applications, such as drug design, or the redesign of new enzymes to perform nonnatural chemical reactions. An essential feature of any protein design algorithm is the ability to accurately model the flexibility that occurs in real proteins. In enzyme design, for example, an algorithm must predict how the designed protein will change during binding and catalysis. In this work we present a large-scale study of 69 protein redesigns that shows the necessity of modeling more realistic protein flexibility. Specifically, we model the continuous space around low-energy conformations of amino acid side chains, and compare it against the standard *rigid* approach of modeling only a small discrete set of low-energy conformations. We show that by allowing the side chains to move in the continuous space around low energy conformations *during* the protein design search, we obtain very different sequences that better match real protein sequences. Moreover, we propose a new protein design algorithm that, contrary to conventional wisdom, shows that we can search the continuous space around side chains with close to the same efficiency as algorithms that model only a discrete set of conformations.

## Introduction

Computational structure-based protein redesign is a promising field with applications for drug design [1], biosynthesis [2], protein∶peptide design [3], and predicting drug resistance [4]. The goal of a structure-based protein redesign algorithm is to search over protein conformations and find the global minimum energy conformation, or GMEC, with respect to a given protein design *model*. The protein design *model* defines both the input to the algorithm and how the redesigned protein can move (the *flexible space*). As input the algorithm takes one or several starting protein structures, an energy function to score the designed proteins, and whether the design search allows amino acid type mutations (a *mutation search*). If mutations are allowed, the protein design algorithm searches protein conformations from multiple sequences to find the amino acid sequence of the GMEC.

**Figure 1:**
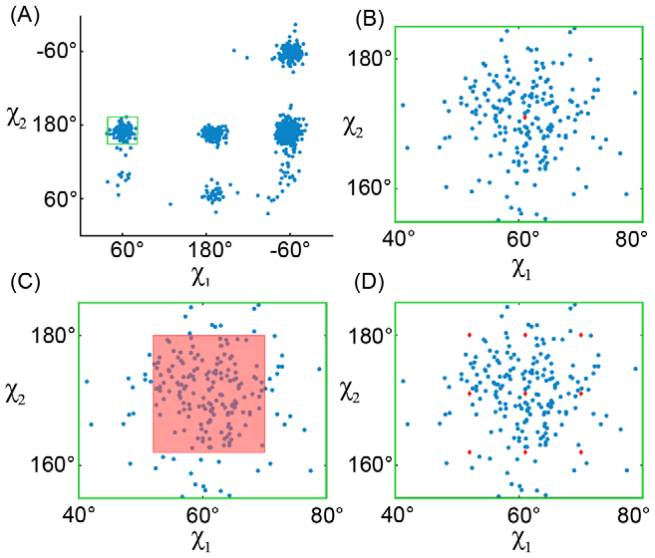
Distribution of Isoleucine in 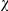-angle space. Isoleucine has two flexible dihedral angles (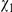 and 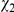 angles) and the ocurrence of isoleucine conformations across a wide set of high-quality structures [38] is plotted here. Panel A shows the entire 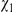 and 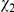 angle space, while panels B, C, and D zoom in on the region specific to one rotamer. (A) The side chains of amino acids commonly appear almost exclusively (blue dots in the plot) within specific regions of their flexible space. (B) In a rigid-rotamer model a single conformation (the red diamond) represents that entire region. (C) In a continuous-rotamer model, a voxel models the continuous region that represents the rotamer. (D) An expanded rotamer model samples additional rigid rotamers near rotamers from the rigid-rotamer model.

Most protein design models limit the flexible space during the search in the interest of computational feasibility. A common protein design model assumes a fixed backbone and only allows the side chains to move among a set of discrete conformations called *rotamers*. Rotamers are determined using theoretical calculations and the empirical observation that the side chains of amino acids in protein structures avoid most of the available conformational space and appear frequently as clusters in 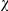-angle space [5] (Figure 1A).

Traditionally, a *rigid-rotamer model* is used for protein design. The rigid-rotamer model represents each empirically-determined side-chain cluster as a single discrete rotamer (usually the modal or mean value of the cluster's distribution is chosen for the rotamer conformation (Figure 1B)). However, protein energetics are sensitive to small changes in atom coordinates, so a single discrete conformation cannot fully describe a continuous region of side-chain conformation space. On the other hand, the *continuous-rotamer model* allows each rotamer to represent a region in 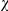-angle space in order to more accurately reflect the empirically-discovered side-chain clusters (Figure 1C). Because both methods use different rotamer models, they obtain different GMECs; we refer to the GMEC when using a rigid-rotamer model, and the continuous-rotamer model, respectively, as the *rigid GMEC* and the *minGMEC*.

**Figure 2:**
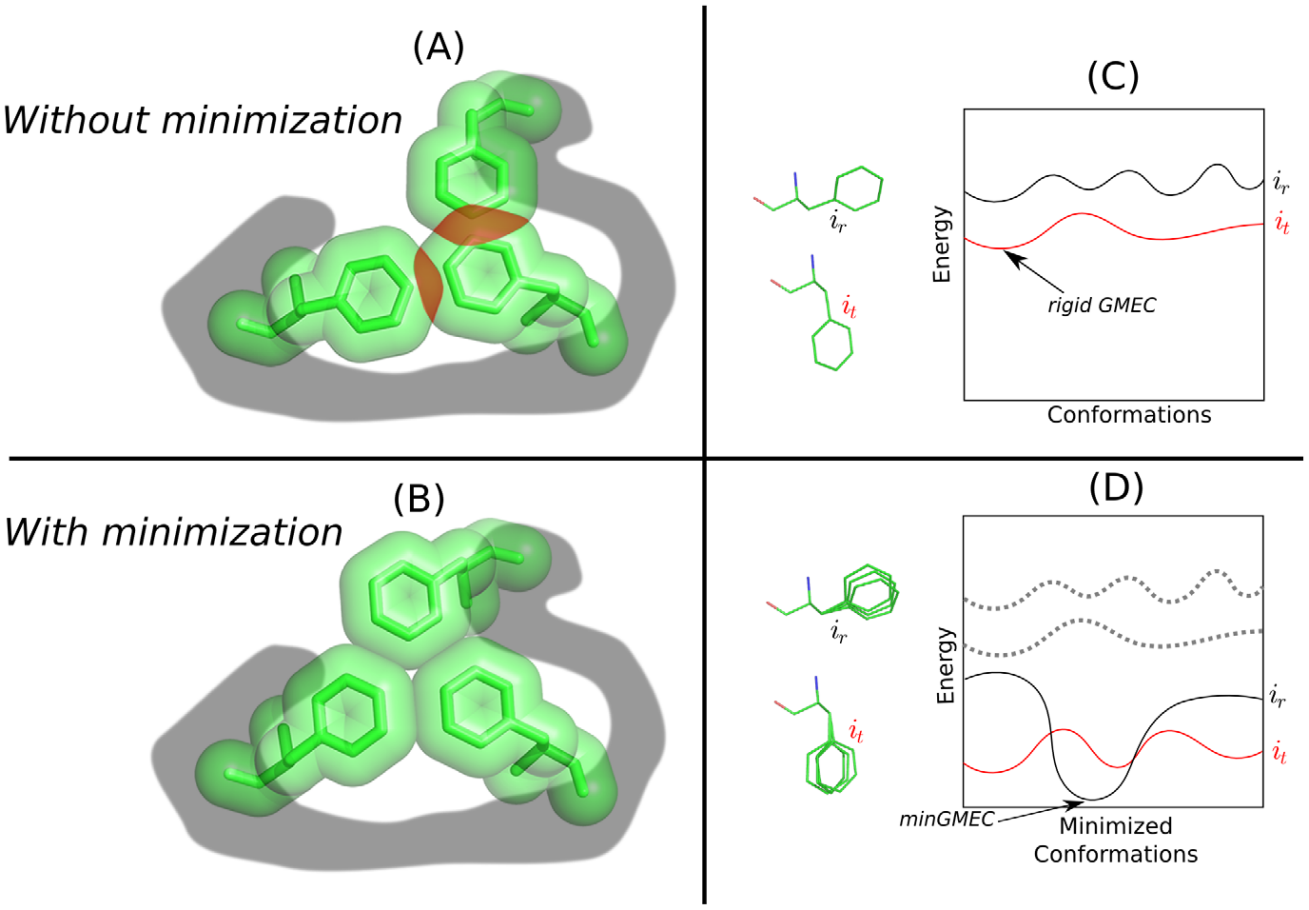
Toy example on the impact of rotamer minimization in protein design and DEE pruning. (A) Many protein design algorithms select a single, discrete conformation to represent each rotamer. The discrete conformation speeds up the computation, but it can result in steric clashes (shown in red). (B) Small changes in 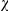-angle space can have profound effects on the energies of interacting rotamers, particularly in the packed core of a protein. The three hydrophobic residues in this toy example can form a well-packed core through small changes in their 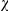 angles in this cartoon. A pruning algorithm like rigid DEE would erroneously prune the clashing rotamers since it does not account for these small changes. (C) If one rotamer, 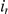, always results in conformations of higher energy than another, 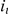, the rotamer 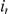 and all the conformations that contain 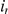 can be pruned. The rigid DEE algorithm [8] prunes rotamers and amino acids that are provably not part of the rigid GMEC. (D) When rotamers can minimize within their specified voxel, rotamers and amino acids that seemed poor in a rigid model might minimize to lower energy conformations than the rigid GMEC. The lowest-energy conformation in this scenario is the minGMEC. The MinDEE algorithm [9] and iMinDEE algorithm can provably prune rotamers in the presence of minimization.

Many protein design algorithms focus on finding the rigid GMEC instead of the minGMEC. These algorithms often try to account for this simplification by allowing side-chain 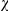 angles to rotate slightly after the rigid search to optimize energy interactions, a process known as *post hoc energy minimization*. This is dangerous because rigid rotamers will often score poorly during a search and be discarded, even though they can potentially minimize to lower energies than the rigid GMEC. The toy example in Figure 2 illustrates how rotamers that are part of a well-packed structure would be discarded by a rigid-rotamer search. Even though a *post hoc* energy minimization of the rigid-rotamer model in this example would result in a low-energy structure, the pre-minimization energy would be so high that this conformation would not be considered for minimization. Thus, rigid-rotamer methods are likely to not even consider the minGMEC as a good candidate structure.

Previous work has shown the benefit of continuously minimizing rotamers [6], [7]. For example, the method described in [7] extends post-hoc energy minimization by allowing rotamers to change during the minimization step. First, a Monte Carlo, rigid-rotamer based algorithm finds a low-energy structure. Next, one residue position at a time, rotamers for that position are continuously minimized, and the lowest energy rotamer is chosen. Thus, the method in [7] is (a) dependent on the rigid-rotamer solution, (b) dependent on the order residue positions are minimized, and (c) does not explicitly allow concerted side-chain movements. In contrast, we use continuous rotamers instead of relying on a rigid-rotamer search. The new design search is no longer over discrete side-chain conformations. Instead, each side-chain rotamer is a continuous region of 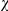-angle space. Therefore, our method is independent of the order in which rotamers are minimized, and allows for coordinated side-chain movements. The use of continuous rotamers guarantees that our protein design search, (i) can find the global minimum energy sequence for continuously minimized side chains, and (ii) never gets stuck in local minima. Our results show the benefits of using continuous rotamers over rigid-rotamer-based models.

In this work we focus on the protein design method dead-end elimination (DEE) because it provably finds the globally optimal solution according to the protein design model. Many protein designs, however, use *heuristic* algorithms instead of provable algorithms. Heuristic algorithms make no guarantees on the optimality of the solution, but they are popular because of their speed. Our results are relevant to these methods as well because the optimal solution computed by DEE provides a bound on the accuracy of all possible heuristic methods. We can therefore measure precisely the limitations of any rigid-rotamer algorithm. The original DEE algorithm (referred to in this paper as *rigid DEE*) finds the GMEC with respect to the discrete rigid-rotamer model by pruning rotamers that provably cannot be part of the rigid GMEC [8]. An advancement of rigid DEE, the MinDEE algorithm [9], [10], addresses the problem of finding the minGMEC by computing an upper and lower bound on the continuous energies of each rotamer and each pairwise rotamer interaction.

In addition to finding lower bounds for each rotamer individually, MinDEE also finds energy bounds for the possible change in energetics that might occur during minimization across the entire protein. The MinDEE pruning criterion prevents the algorithm from using a rotamer 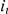 to prune a rotamer 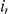 if 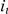 could potentially perturb the other minimizing side chains during its minimization to make it a higher energy rotamer than 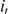 (Figure 2). Even though MinDEE is a powerful technique that prunes the design conformation space by orders of magnitude, the range of potential minimization perturbations that MinDEE considers results in unrealistically loose bounds that bracket each energy interaction. These bounds represent theoretical worst cases which reduce MinDEE's capacity to prune. Therefore, MinDEE's pruning power is significantly weaker than rigid DEE.

MinDEE is an integral part of the 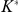 algorithm [2], [9], [11], an ensemble-based algorithm that estimates the binding constant of a protein-ligand complex through a provably-accurate approximation of the partition function. 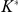 was used prospectively in drug design [1], enzyme redesign [2], protein∶peptide design [3], and drug resistance prediction [4], all with experimental validation. 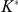 approximates the partition function by evaluating only the low energy conformations that carry the largest weight in the Boltzmann-weighted partition function. The MinDEE algorithm is essential for 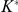, since MinDEE prunes the majority of conformations that cannot minimize into low energy conformations, and therefore need not be considered by 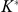. Therefore, improvements to the MinDEE criterion and algorithm directly improve the efficiency of MinDEE/A* and the 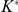 algorithm.

In this work we show that when a protein design algorithm uses a continuous-rotamer model, the algorithm is able to find the minGMEC, which is often a much lower energy sequence than the rigid GMEC. Specifically, we show that the MinDEE algorithm is able to find lower energy sequences than those found by rigid DEE in 66 out of 69 proteins from the PDB. We also show that trying to find the minGMEC by increasing the number of rotamers in the rigid-rotamer model (Figure 1D) is often impractical, and still fails to find the minGMEC in most cases. In addition, we propose a simplified and improved alternative to MinDEE, which we call *iMinDEE*. iMinDEE uses a new technique that we call *Greedy Estimation of Minimization* (GEM), which allows iMinDEE to reduce the search space by orders of magnitude when compared to MinDEE. iMinDEE and MinDEE are mathematically guaranteed to compute the same results, and to check this is true, we ran both algorithms and obtained identical results. Finally, we used *native sequence recovery*, a commonly used metric to evaluate protein design algorithms, to show that continuous rotamers result in more biologically accurate protein redesigns. We tested how well the sequences of both the minGMEC and the rigid GMEC recapitulated the native protein sequence and found that iMinDEE significantly improves native sequence recovery over rigidDEE.

## Results

In this work we focus on the importance of using continuous rotamers instead of rigid rotamers in protein design. First, we establish that protein design searches that use continuous rotamers find sequences lower in energy than those using rigid rotamers. Next, we present an improved and simplified DEE pruning criterion that makes continuous-rotamer protein design more computationally feasible.

### Impact of continuous rotamers on protein design

In this section we first describe the original rigid DEE [8] and MinDEE criteria [9], and then show an experimental comparison of the two methods. This comparison shows that MinDEE provides a substantial advantage over rigid DEE in computing low-energy sequences. Finally, we compare a rigid-rotamer protein design search using an expanded rotamer library against MinDEE with a standard rotamer library.

#### Rigid DEE criterion

The rigid DEE criterion [8] prunes rigid rotamers that cannot be part of the GMEC for a given protein design system. To prune a candidate rotamer, rigid DEE finds a competitor rotamer at the same residue position that can always provide a lower energy than the candidate rotamer. Let the internal energy of rotamer 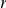 at residue position 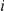, 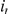 be 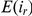, the pairwise energy between rotamers 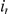 and 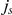 be 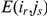 and 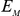 be the template energy (i.e. the energy of the backbone atoms and side chain residues that are not allowed to move or mutate). The protein design system can be represented as a rotamer vector, 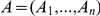, which is an assignment of a rotamer 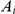 at each design position 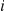. Then we define the total energy 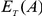 of the system 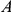:
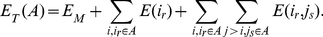
(1)


The dead-end elimination criterion states that for a rotamer 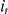, if there is a rotamer 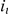 such that:
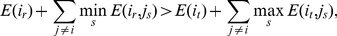
(2)then 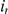 is provably not part of the GMEC, and can therefore be pruned. Rigid DEE prunes rotamers in sequential iterations; the pruning of a rotamer at position 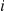 in one iteration might enable the pruning of a rotamer at position 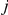 in the next iteration.

#### MinDEE

The MinDEE criterion [9], [12] extends the rigid DEE criterion to provably prune only rotamers that cannot minimize to the minGMEC. MinDEE treats rotamers as a continuous range of conformations inside a voxel 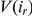 over the space defined by movements up to 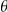 degrees from the modal value. MinDEE sets bounds for the energy of each voxel through a maximum energy, 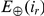, and a minimum energy, 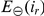 to be used for pruning. In the case of pairwise energies, MinDEE sets bounds for the minimum and maximum interaction energies between residues 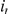 and 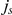 within the space 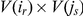: 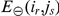 and 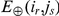 respectively. When energy minimization is not allowed, the energy of a fully-assigned rotamer vector 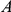, 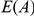, can be computed as a sum of independent, individual terms (Eq. (1)). When energy minimization is allowed, however, the minimized energy of 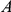, 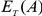, cannot be pairwise-decomposed, since the minimization of one rotamer within its voxel might alter how the remaining rotamers minimize (i.e. a domino effect). 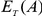, however, can be bounded by the sums of maxima and minima [9], 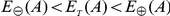:
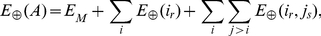
(3)

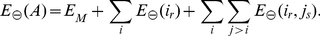
(4)


In order to prune rotamers, possible perturbations that minimization may cause in the rest of the system must be accounted for. MinDEE accounts for possible side-chain rearrangements during minimization by including the maximum range terms: 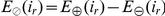, 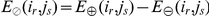. The MinDEE criterion for pruning [9] is:
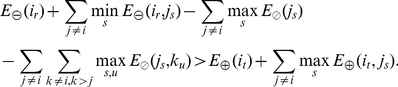
(5)If Eq. (5) holds for rotamers 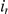 and 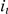, then rotamer 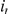 is provably not part of the minGMEC.

#### MinDEE/A*

MinDEE prunes rotamers that are provably not part of the minGMEC, and then the A* [9], [13] algorithm is used to enumerate rotamer vectors in order of the lower bound on their energies. During the A* search, each rotamer vector is minimized and the A* enumeration stops when the lower energy bound of the enumerated conformation is higher than the lowest minimized energy.

#### Energy comparison between rigid DEE and MinDEE

**Figure 3:**
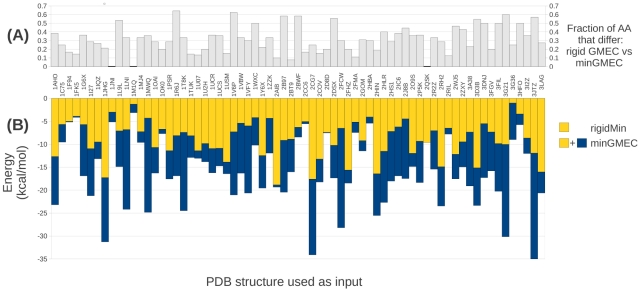
Rigid GMEC vs. minGMEC. (A) Fraction of the redesigned residues that had different amino acids (AA) between the rigid GMEC and the minGMEC. In 66 out of the 69 cases the minGMEC and the rigid GMEC have different sequences. The three systems where the minGMEC has the same sequence as the rigid GMEC are marked with a bold line at zero (2QSK, 1M1Q, and 1JNI). (B) Energy of the minGMEC vs. energy of the rigidMin (the *post hoc* minimization of the rigid GMEC), relative to the energy of the rigid GMEC, which is set to zero for each system. In 68 of 69 cases the energy of the minGMEC is lower than that of the rigidMin. For 2QSK the rotamers of the rigid GMEC are the same as the rotamers of the minGMEC, and, therefore, the energy of the rigidMin is the same as the energy of the minGMEC. The energy of the minGMEC is shown in yellow + blue bars, while the yellow color by itself shows the energy of the rigidMin. The results of this figure are identical for iMinDEE and MinDEE since both algorithms provably find the minGMEC.

Both the rigid GMEC and the minGMEC were computed for 69 protein core redesigns. As a postprocessing step, the rigid-GMEC conformation was energy minimized to make the comparison fair, since many programs that use rigid rotamers minimize the rigid GMEC after rigid DEE. We will refer to the *post hoc* energy minimized rigid GMEC as the *rigidMin*. Figure 3 shows a comparison between the energy of the minGMEC and the rigidMin, normalized to the energy of the rigid GMEC for 69 design runs. In 68 of the 69 design runs the minGMEC had a lower energy than the rigidMin, with an average energy difference of 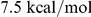 (standard deviation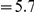) and a maximum energy difference of 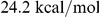. In only one design case, antiviral lectin scytovirin from *Scytonema varium* (PDB id: 2QSK) are the minGMEC and the rigidMin the same, with the same minimized energy and the same sequence. Furthermore, in 66 of the 69 design runs the minGMEC was different from the rigid GMEC. We evaluated the *sequence distance*, the percentage of designed residues that differ in their amino acid type between the rigid GMEC and the minGMEC, and found a sequence distance average of 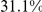 (standard deviation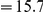). The maximum sequence distance is 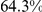. For two design runs, Cytochrome C from *Shewanella oneidensis* (PDB id: 1M1Q), and NapB from *Haemophilus influenzae* (PDB id: 1JNI), the minGMEC and the rigid GMEC have the same sequence, but different rotamers and therefore different energies. Both of these designs are small: only 4 redesigned residues for 1M1Q and 5 for 1JNI.

To further illustrate these results we present the results from a representative design run, the run for Ribonuclease from *Streptomyces aureofaciens* (PDB id: 1LNI). The rigid GMEC (computed with DEE/A*) has an energy of 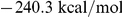, the rigidMin has an energy of 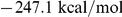, and the minGMEC (computed with MinDEE/A*) has an energy of 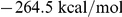. Five amino acids differ between the minGMEC and the rigid GMEC: the minGMEC has D33, Y52, R69, M70, and F89; the rigid GMEC has N33, H52, N69, T70, and H89. If the rotamers from the minGMEC are returned to their rigid, modal values, the energy of this conformation is 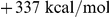, over 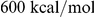 above the minGMEC. This illustrates how a method that relies on rigid rotamers, followed by a *post hoc* minimization step, can miss the minGMEC.

These results clearly show that if minimization is not included *during* the search, the true lowest-energy sequences are missed in almost every case and in many cases the minGMEC has a much different sequence than the rigid GMEC. This also shows that energetically favorable rotamers are pruned because of the inability of rigid rotamers to make small spatial adjustments. More importantly, this means that wet lab experiments based on rigid DEE results, even with *post hoc* energy minimization, will not test the sequences that are predicted to be the best by the energy model.

#### MinDEE vs. an expanded rotamer library

A seemingly simpler alternative to MinDEE is to increase the granularity of the rotamer library and use the rigid DEE algorithm. In practice, however, this is hard because the precomputation of pairwise interactions, the rigid DEE pruning stages, and the A* conformational search are computationally expensive for side chains with 3 or 4 degrees of freedom. For example, consider a rotamer library that is expanded by adding all rotamers with dihedrals 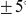 and 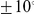 from rotamers in the original library. In such a library an arginine residue that originally had 34 rotamers would increase to 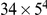 rotamers. In this scenario, a pairwise computation between two arginine residues must consider 450 million pairs.

**Figure 4:**
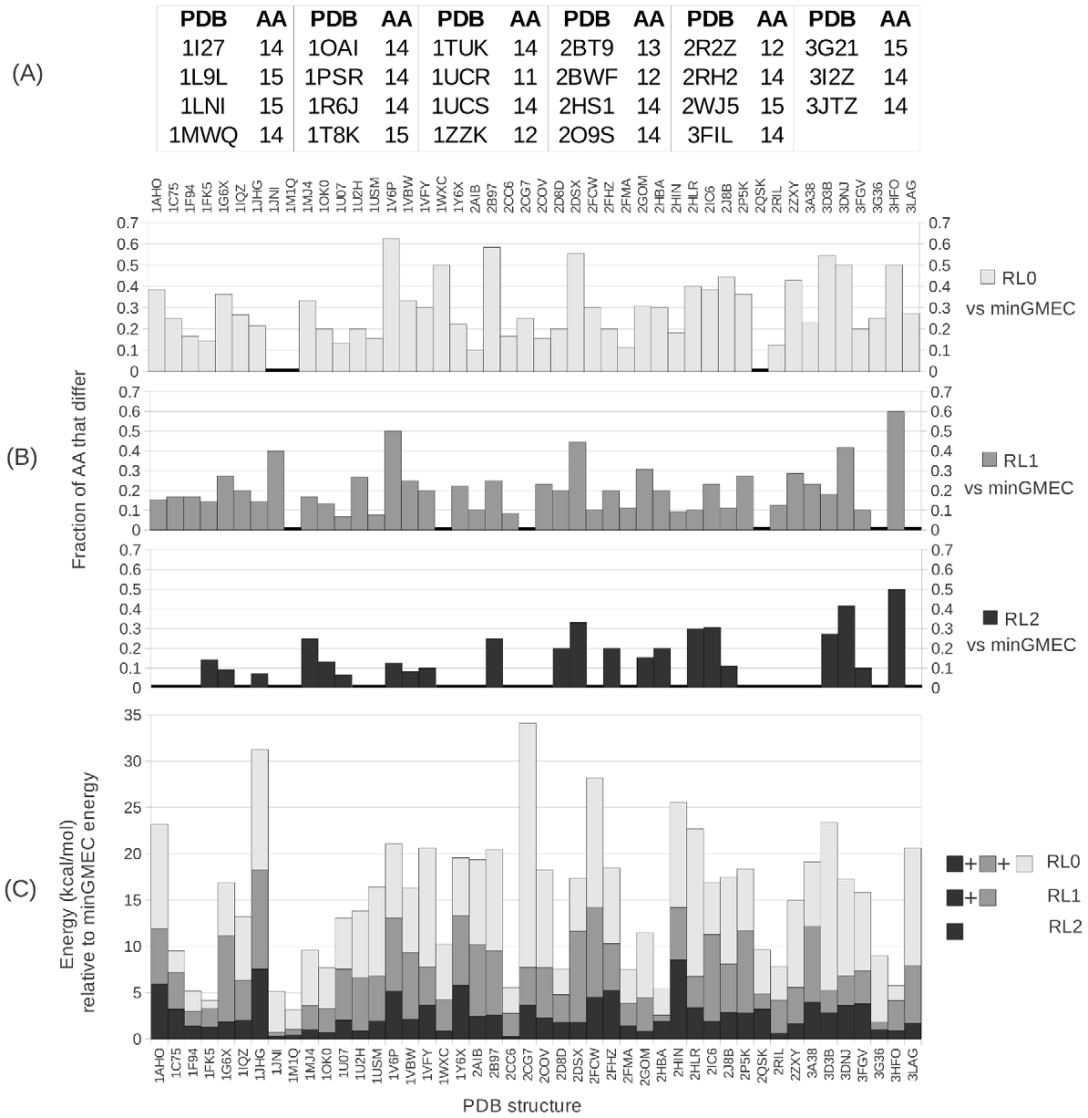
The minGMEC vs. rigid DEE with an expanded rotamer library. Two expanded rotamer libraries were used, RL1 and RL2, and they were compared against the standard rotamer library (RL0). (A) Redesigns that failed for rigid DEE using rotamer library RL2 because of the library's large size. AA: The number of mutable amino acids. (B) Fraction of the amino acids that are different between the minGMEC of MinDEE and, respectively: the rigid GMEC of RL0 (light grey), the rigid GMEC of RL1 (grey), and the rigid GMEC of RL2 (dark grey). Those designs where the sequence of the minGMEC and the sequence of the rigid GMEC are the same are marked with a bold line at zero. (C) Energy of the rigid GMEC of RL0 (light grey + grey + dark grey) vs. the rigid GMEC of RL1 (grey + dark grey) vs. the rigid GMEC of RL2 (dark grey), relative to the energy of the minGMEC, which is set to zero for each system.

To overcome this rotamer explosion, some protein design protocols [14], [15] add more rotamers by altering only the 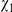 or 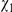 and 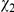 angles by 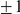 standard deviation 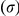. We tested this approach by building two expanded rotamer libraries from the Richardson's Penultimate Rotamer Library: RL1, a rotamer library where new rotamers are added by varying each rotamer's 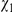 angle by 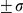; and RL2, an extension where rotamers are added by varying both 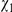 and 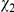 by 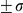. We then compared the rigid GMEC of the original rotamer library (denoted as RL0), RL1, and RL2 against the minGMEC for each system. The energetic and sequence results for these rotamer libraries are shown in Figure 4.

Figure 4 shows results for only 46 proteins, much less than the 69 shown in Figure 3, because rigid DEE with rotamer library RL2 failed for 23 of them. The results for the 46 proteins that did finish for RL2 show that on average the RL0 rigid GMEC is 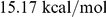 higher in energy than the minGMEC; RL1 is 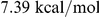 above the minGMEC; and RL2 is 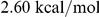 above the minGMEC. The amino acid sequences also vary between the expanded rotamer libraries and the minGMEC, with an average difference of 28% for RL0, 18% for RL1 and 10% for RL2.

**Figure 5:**
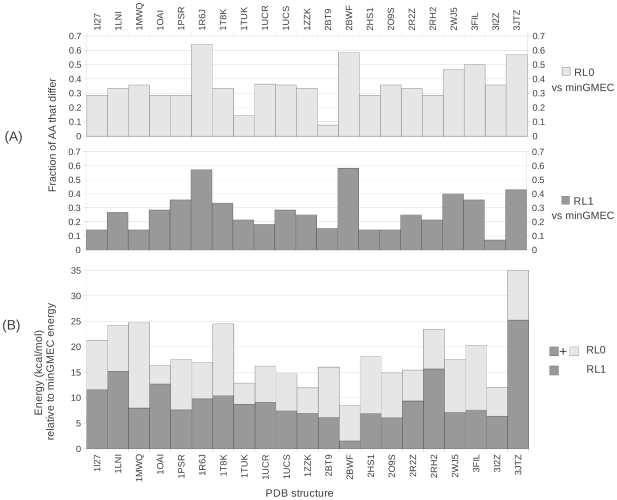
The minGMEC vs. rigid DEE with an expanded rotamer library for the systems that failed with rigid DEE using rotamer library RL2. These results compare the standard rotamer library (RL0) against an expanded rotamer library, RL1. (A) Fraction of the amino acids that are different between the minGMEC of MinDEE and, respectively: the rigid GMEC of RL0 (light grey), and the rigid GMEC of RL1 (grey). (B) Energy of the rigid GMEC of RL0 (light grey + grey) vs. the rigid GMEC of RL1 (grey), relative to the energy of the minGMEC, which is set to zero for each system.

The remaining 23 systems ran out of memory on the rigid DEE runs with rotamer library RL2, either in the DEE stages, or in the A* stage. This ocurred because the rotamer library RL2 is too large, even though our protein core redesigns are restricted to at most 15 mutable residues. Two redesigns, 1L9L and 3G21, both with 15 redesigned residues, failed for both RL1 and RL2 rotamer libraries. The results for the 21 systems that failed with rotamer library RL2 but completed with rotamer library RL1 are shown in Figure 5.

### Greedy Estimation of Minimization (GEM)

The MinDEE algorithm is guaranteed to find the GMEC when searching over continuous rotamers, which we call the minGMEC. To efficiently prune and search over continuous-rotamer conformations, the MinDEE algorithm computes lower and upper bounds on the pairwise energies of continuous rotamers (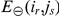 and 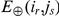, as defined above). In practice, however, these maximum and minimum bounds can be very loose. This results in a large gap between the maximum and minimum terms, which consequently makes the 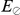 terms in the MinDEE pruning criterion (Eq. 5) very large. For example, a pair of neighboring tryptophan rotamers might have a maximum energy within a voxel of 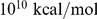, but these can minimize and form favorable stacking to an energy of 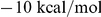. These large 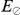 terms make it difficult to prune rotamers, resulting in much less pruning than rigid DEE.

In this section we present a new criterion and algorithm, iMinDEE, which can prune rotamers much more efficiently than MinDEE and is still guaranteed to find the minGMEC. iMinDEE obtains improved pruning by removing the need to define maximum bounds on continuous-rotamer energies, which eliminates the large 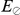 terms from the pruning criterion. Remember that the 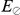 terms from the MinDEE criteria were needed to account for all possible side-chain rearrangements that could occur during protein minimization. Instead of accounting for all potential side-chain rearrangements, iMinDEE greedily estimates how much minimization can actually occur.

We refer to the overall technique that iMinDEE uses to prune rotamers as *Greedy Estimation of Minimization (GEM)*. The basis behind GEM is to greedily assume that protein minimization occurs independently for each rotamer pair. Rotamers are initially pruned based on this assumption, and the A* algorithm finds the best conformation in the remaining (unpruned) conformational search space. After this first run, we can check whether the assumption was wrong and if the minGMEC was pruned. Remarkably, if the minGMEC was pruned, we can provably refine our initial assumption to obtain a new pruning criterion that is guaranteed to recover the minGMEC, and the algorithm will run at most one more time.

#### Interval MinDEE

We propose an improved algorithm for continuous-rotamer pruning called *Interval MinDEE (iMinDEE)* which eliminates the need for defining maximum bounds on the energy terms of rotamers. Instead, iMinDEE uses an interval term, 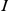, that accurately bounds the minimization that can occur within the protein. This allows for much tighter energy bounds than the MinDEE method and therefore much more pruning.

To account for side-chain minimization the iMinDEE algorithm computes lower bounds on the internal and pairwise energies of continuous rotamers. Each continuous rotamer represents a continuous set of side-chain conformations (i.e., a set of 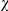 angles) that can be interpreted as a voxel in 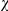 angle space. Consider a pair of continuous rotamers, 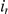 and 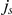. The pairwise energy 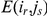 of 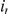 and 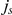 varies as 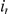 and 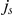 each take on conformations defined by the parameter space of their voxel. To bound these pairwise energies, iMinDEE calculates the lowest-energy conformation for a rotamer pair when no other side chains are present. Unfortunately, once additional residues are added to the protein, and the entire conformation is minimized, it is no longer guaranteed that a single rotamer pair will maintain its lower bound conformation. Thus, during the design search when calculating the energy of a full protein conformation, the actual energy of a rotamer pair will always be higher than the precomputed low-energy bound. The interval term, 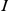, in the iMinDEE pruning criteria accounts for this energy difference for all rotamer pairs.

We now define the interval term. Let 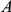 be any valid rotamer assignment. Let 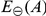 be the low-energy bound of rotamer assignment 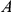 and let 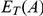 be the total minimized energy of 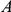. Let 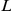 be the rotamer assignment with the lowest energy bound and let 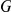 be the rotamer assignment of the minGMEC. By definition, 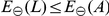 and 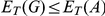. We define the interval 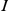 as:
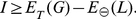
(6)We now define the iMinDEE criterion:
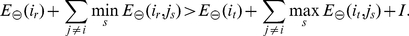
(7)

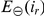
 is the lower bound on the energy of rotamer 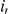, and 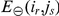 is the lower bound on the pairwise energy of rotamers 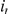 and 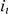, as defined in the *MinDEE* section above. If Eq. (7) holds, then 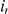 is provably not part of the minGMEC.

**Proposition 1.** *When* *Eq. (7)* *holds, rotamer* 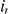 *can be provably pruned from the search space because it cannot be part of the minimized global minimum energy conformation (minGMEC).*

The proof for Proposition 1 is given in Text S1.

The smaller the value of 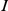, the greater the pruning by iMinDEE. However, determining the optimal value of 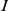 would require computing the optimal rotamer assignment 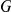, so finding the optimal 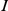 is as hard as solving the problem of finding the minGMEC. Instead, we find an approximation for the optimal value of 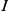 as outlined below.

#### Greedy estimation of a good approximation for 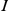


In this section we detail the GEM technique to find a valid approximation for the optimal value of 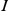. The algorithm is sound, must only be repeated at most once, and guarantees that iMinDEE finds the minGMEC. First, we choose an initial approximation for 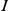, called 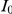 (in our implementation we found setting 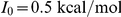 worked well). Next, we prune the rotamer library using the iMinDEE criterion (Eq. (7)) substituting 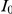 for 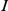. After pruning, we use A* to enumerate protein conformations in order of their lower energy bound and compute the minimized energy of the enumerated conformations. Let 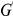 be the lowest energy conformation found during the enumeration. Since 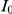 was only an initial guess for 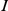, it is possible that the optimal value of 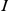 is greater than 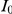. If that is the case, then 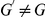, where 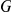 is the minGMEC that we are trying to find. To check the validity of 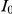 we define a second approximation to 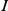 called 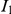:
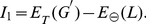
(8)Using the proposition below, we can determine whether 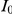 was a valid approximation for 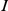. If it was not, then 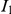 is guaranteed to be a valid approximation for 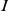. Finally, we can repeat the pruning and A* steps using 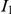 instead of 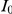, and are guaranteed to find the minGMEC during this A* search.

**Proposition 2.** *If* 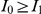 *then* 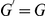 *and the search can stop; otherwise the search must be repeated once using* 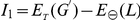 *to find the minGMEC.*

*Proof.* First consider if 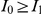. Then using the definitions of 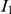 and the fact that 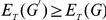:
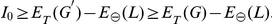


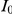
 satisfies Eq. (6), which means that the pruning criterion is valid and 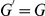. Now consider if 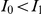. In this case the pruning criterion used was not correct so the design can be rerun using 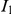 instead of 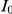. By definition we know that 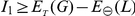 so as in the first case the pruning criterion is valid and 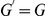.

**Figure 6:**
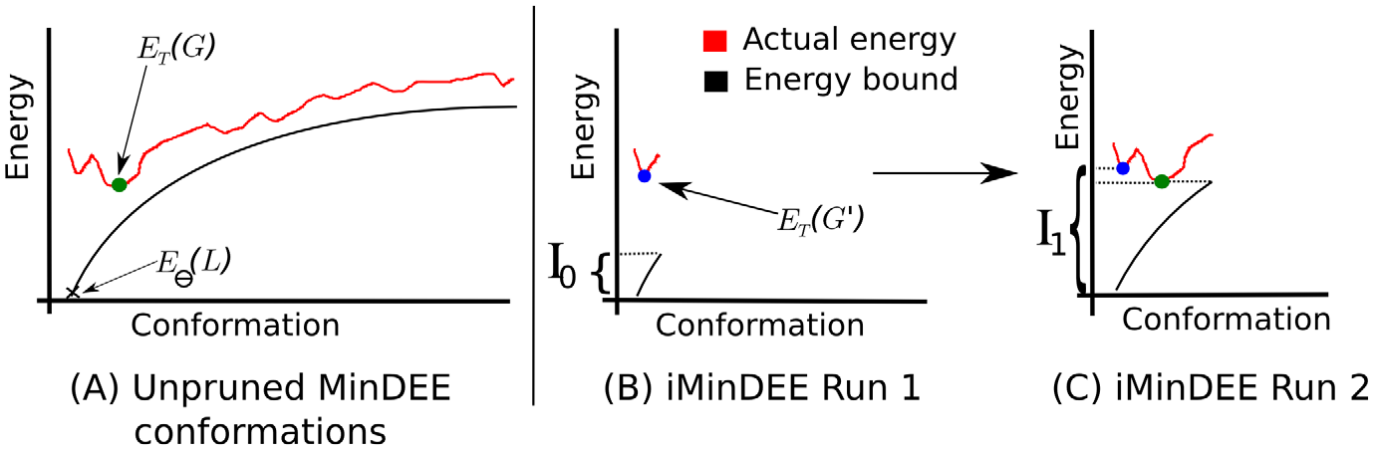
iMinDEE algorithm illustration. The A* branch-and-bound algorithm completely searches the conformation space and enumerates conformations in order of their low-energy bound. Because the search is complete, a large conformational search space can be computationally infeasible for A*. Therefore, a pre-A* pruning of the conformational search space with the MinDEE algorithm or iMinDEE algorithm can make the A* search feasible. (A) The entire MinDEE conformation space in the order that the A* algorithm would enumerate the conformations. A* enumerates conformations until it can prove the minGMEC (denoted as 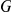) has been found, but unpruned high energy conformations slow down the search. The first conformation enumerated by A*, corresponding to the conformation with the lowest energy bound, is denoted 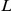, and the lower bound on its energy is 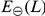. The minGMEC, 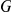, is marked by a green dot and its energy is 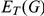. (B) Instead of MinDEE, we can use iMinDEE to prune conformations with energy bounds that are higher than the lowest energy bound by more than the initial 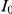 value. We then select the lowest minimized energy found so far (i.e. as opposed to lowest energy *bound*) and use that to compute the 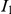 value. The conformation with the lowest minimized energy is denoted 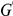 with a blue dot and its energy is 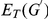. (C) The iMinDEE search is repeated if 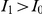. Since 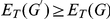, 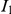 meets the condition of Eq. (6), and the search will not need to be repeated again. By setting 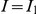, we can use the iMinDEE criterion (Eq. (7)) to prune rotamers, and the iMinDEE algorithm will provably find the minGMEC.

**Figure 7:**
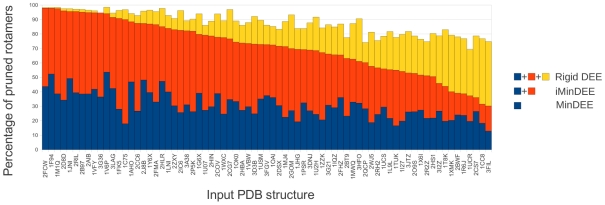
Comparison of rotamer pruning with rigid DEE, MinDEE and iMinDEE. For each tested protein, this chart shows what percentage of rotamers were pruned by each criterion. In all cases pruning with rigid DEE pruned at least as much as iMinDEE, and pruning with iMinDEE was significantly better than MinDEE.

Figure 6 illustrates how the entire algorithm works. The algorithm repeats at most once and is guaranteed to find the minGMEC. Even though iMinDEE must go through two phases of pruning and A* enumeration, this is a constant factor increase in runtime, and in practice iMinDEE is still much faster than MinDEE. By removing the maximum energy bounds (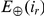 and 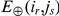 in Eq. (5)) from the MinDEE criterion, the iMinDEE criterion is able to prune significantly more than MinDEE (See Figure 7).

### Analysis of iMinDEE

iMinDEE is mathematically guaranteed to compute the same result as the original MinDEE, but can do so much more efficiently. To show the benefit of our approach, we implemented iMinDEE and applied it to the 69 protein core redesigns. We show that iMinDEE significantly reduces the conformation search space compared to the original MinDEE criterion. In many cases iMinDEE is nearly as efficient as rigid DEE, while still guaranteeing to compute the minGMEC. Finally, we analyze the meaning and impact of the interval term, 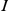, in the iMinDEE criterion.

#### Comparison between rigid DEE, MinDEE, and iMinDEE pruning

The protein design runs analyzed with rigid DEE and MinDEE in the previous section were conducted using the iMinDEE criterion. Figure 7 shows a comparison between the percentage of rotamers pruned by rigid DEE, iMinDEE and MinDEE. In all cases pruning is significantly higher for iMinDEE compared with MinDEE, and in some cases iMinDEE pruning is as efficient, or nearly as efficient, as rigid DEE. We again select the mid-ranking (in terms of iMinDEE pruning) Ribonuclease (1LNI) design run to look at the results in more detail. The Ribonuclease structure has 15 residues with a SASA of less than 5% that were selected as mutable. This results in a search space of 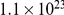 conformations. The MinDEE algorithm prunes 40% of all rotamers, which reduces the number of conformations to 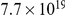. In contrast, iMinDEE prunes 83% of all rotamers and reduces the search space to 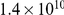. Rigid DEE prunes 93% of all rotamers and reduces the search space to 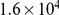. This means that the remaining search space that is input into A* is 5.5 billion times smaller when iMinDEE is used than when MinDEE is used.

Rigid DEE is not directly comparable with MinDEE/iMinDEE because, as Figure 3 shows, it almost always finds a different (and worse) answer than MinDEE. We feel, however, that a comparison of pruning is necessary since rigid DEE is the standard in the field, and potential adopters of iMinDEE might feel reluctant to migrate if it results in considerable performance penalties. Results of the pruning comparison show that in most cases iMinDEE prunes with close to the same efficiency as rigid DEE while maintaining the guarantees of MinDEE.

#### Analysis of the interval term

The interval term 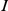 in the iMinDEE pruning criteria accounts for potential side-chain rearrangements that can occur when one rotamer is changed to another rotamer. Since the optimal value of 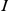 cannot be computed efficiently, the iMinDEE algorithm uses the computed value 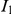 (Eq. (8)) during the final round of pruning. When we determine that a design system has a high 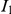 value, by definition this means that the difference between the rotamer pair bounds and the actual minimized energy of the protein system is large. Thus, the 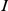 value is intrinsic to each design system, and is a good indication of whether the system can be tractably designed or not.

**Figure 8:**
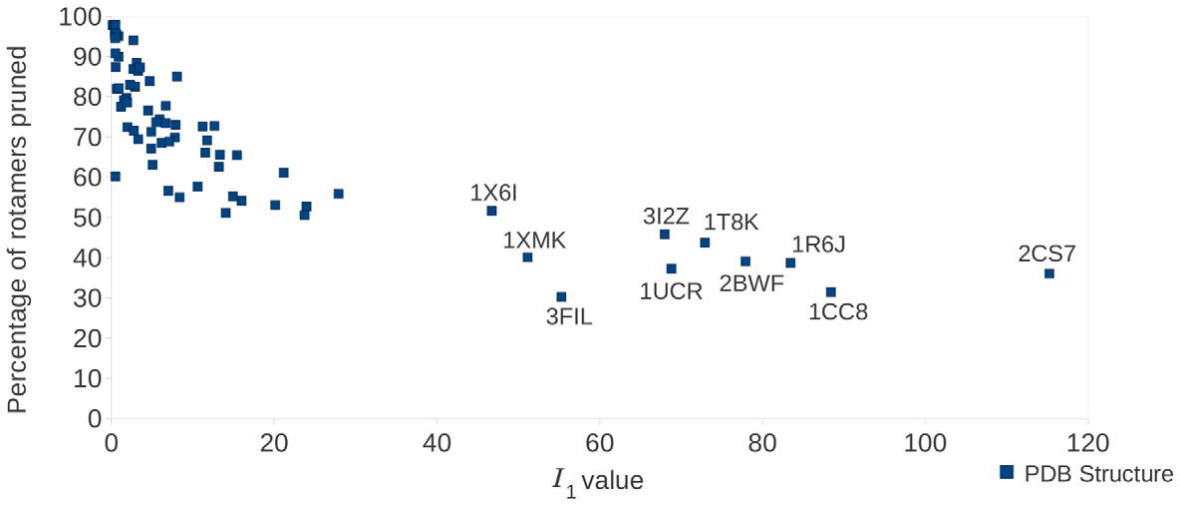
Pruning vs 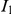 value. Most systems have small 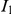 values. Some outliers have larger 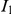 values, and in consequence, iMinDEE loses pruning efficiency in these systems.

Figure 8 shows the relationship between 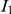 and pruning power of iMinDEE for our protein design test set. Clearly, as the value of 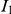 decreases iMinDEE can prune more rotamers. Ten 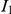 *outlier* systems that had pruning levels at or below 50% are labeled in Figure 8 (PDB ids: 1X6I, 3FIL, 1UCR, 3I2Z, 1T8K, 2BWF, 1R6J, 1CC8, 1XMK, and 2CS7). Since the pruning for these 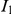 outliers was low, our iMinDEE/A* implementation was unable to compute the minGMEC for four of these runs (1X6I, 1XMK, 1CC8, and 2CS7). Because we were not able to compute the minGMEC for these four runs, they are not included in Figures 3, 4, and 5. These four runs also ran out of memory in the rigid DEE/A* runs with rotamer library RL2, and the runs for 1X6I and 2CS7 ran out of memory with rotamer library RL1.

**Figure 9:**
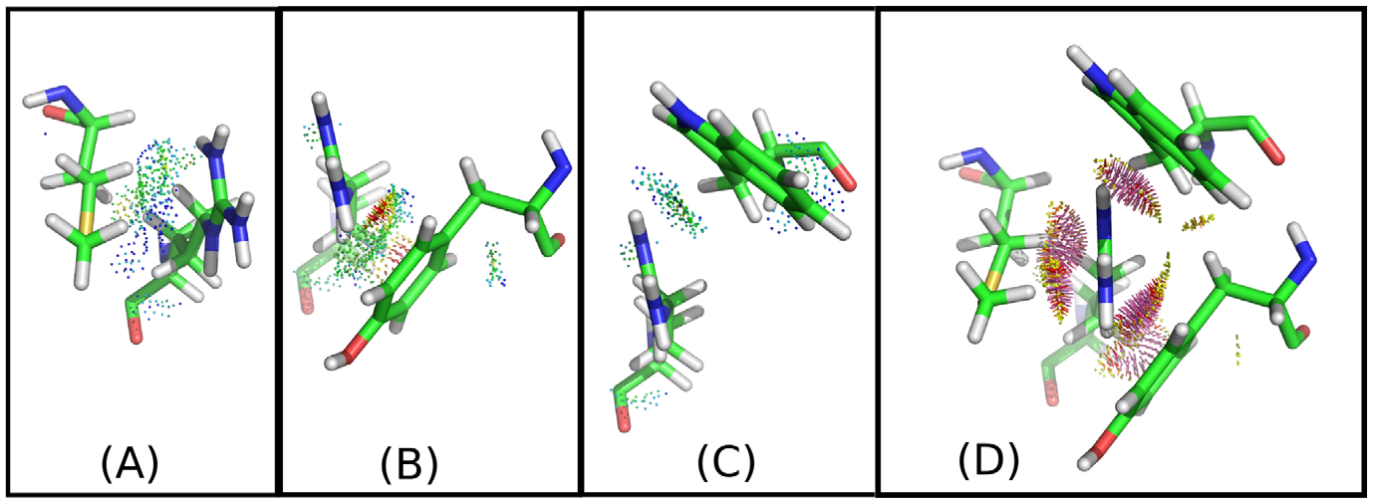
iMinDEE predicts residues Trp3 (rotamer 3), Tyr11 (rotamer 1), Met40 (rotamer 8), and Arg44 (rotamer 15) in the structure of the PhtA histidine triad domain (PDB ID: 2CS7) to achieve a low-energy conformation. iMinDEE precomputes low-energy bounds between all pairs of possible rotamers in structure 2CS7. This figure illustrates the lower bound between the pairs (A) Met40 and Arg44, (B) Trp3 and Arg44, and (C) Tyr11 and Arg44. Favorable vdW contacts are shown in green and blue dots, and a small steric overlap is shown in red in pane (C). All of these pairs have favorable, low energies and iMinDEE predicts all conformations containing the 4 rotamers shown in this chart to be among the lowest energy structures. (D) When all four are placed in the same conformation, however, the result is a biophysically impossible steric clash, shown by red and purple dots.

A close examination of all ten 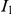 outlier structures showed a common pattern: in the absence of neighboring rotamers, rotamer pairs would minimize into conformations that were incompatible with other rotamers when all rotamers were minimized together. Interestingly, eight of these structures have trouble spots where a single rotamer is responsible for most of the gap between the energy lower bound and minimized energy. To analyze this graphically (Figure 9) we chose the most outlying design run, which was of the *S. pneumoniae* PhtA histidine triad domain (PDB id: 2CS7). This structure has one trouble spot involving Arg44 and its pairwise interactions with residues Trp3, Tyr11, and Met40. Arg44 clashes with each of its neighbors in its rigid-rotamer conformation, but each pairwise clash can be solved through minimization. When all rotamers are present, however, solving the clash with one pair results in Arg44 moving to clash with another rotamer. The result is that iMinDEE will enumerate all the conformations that contain the four mutants, because they have a good lower bound, but none of them can result in a good global conformation because the Arg44 clashes with all of its neighbors when they are all present. This suggests that using a higher-order bounds computation might be able to resolve this particular case.

When we ran rigid DEE with rotamer library RL2 (Section “MinDEE vs. a finer rotamer library,” above), the design runs for all of the ten 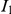 outlier systems (1X6I, 3FIL, 1UCR, 3I2Z, 1T8K, 2BWF, 1R6J, 1CC8, 1XMK, and 2CS7) failed to complete because they ran out of memory. In addition to the ten 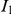 outliers, rigid DEE/A* with RL2 could not compute the rigid GMEC in 17 other cases (Figure 4A) and these systems often have high 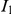 values. Therefore, rigid DEE with an expanded rotamer library is both unable to reach the energy of the minGMEC (Figure 4), and unable to perform better than iMinDEE even in cases where iMinDEE has little pruning. Since iMinDEE was able to compute the minGMEC for the 23 systems that failed with rigid DEE/A* and RL2 (Figure 4A), this further emphasizes the benefit of iMinDEE over expanded rotamer techniques.

#### Native sequence recovery using continuous rotamers

There is evidence suggesting that the sequences of native proteins optimize the stability of their backbone structure [16]. Using this hypothesis, a common way to evaluate protein design algorithms is to see how well the low-energy sequence found by the algorithm compares with the native protein sequence. While it is most likely true that some residues are optimized for function instead of stability [17], native sequence recovery still remains a valuable tool to determine the biological relevance of new protein design algorithms. Therefore, to analyze the benefits of continuous rotamers for protein design, we compared the native sequence recovery of iMinDEE with that of rigid DEE.

For the native sequence recovery tests, we chose to design those proteins from our initial test set of 69 proteins that had no co-factors or non-amino acid ligands interacting with core residues. It is expected that side chains interacting with co-factors or ligands are involved in binding and catalysis, and are not necessarily optimized for the unbound structure. Therefore, sequence recovery is not applicable to these functional residues, because their identity is determined by more than just *apo* energetic structural stability. 43 protein structures remained after removing those with interacting co-factors and ligands, which resulted in a total of 527 residue positions to be redesigned. We redesigned each protein system with both rigid DEE and iMinDEE, using the same energy parameters for both algorithms. We then compared both the rigid GMEC computed by rigid DEE, and the minGMEC computed by iMinDEE, vs. the native sequence.

**Figure 10:**
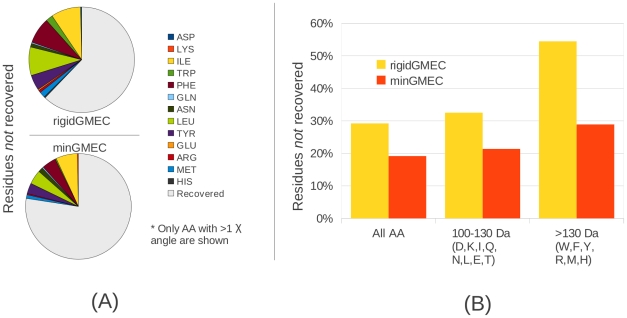
Summary of native sequence recovery results. The recovery of native amino acid sequence by rigid DEE (the rigid GMEC) and by iMinDEE (the minGMEC) are shown. (A) Summary of amino acid side chains that contain more than one flexible dihedral angle (asp, lys, ile, trp, phe, gln, asn, leu, tyr, glu, arg, met, and his) that were *not* recovered by the rigid GMEC (pie chart above) and the minGMEC (pie chart below). For comparison, the recovered amino acids with more than one flexible dihedral angle are shown in grey. Residues that were not recovered are colored by their amino acid type. (B) Percentage of residues *not* recovered by the rigid GMEC (yellow) and the minGMEC (orange), categorized by amino acid mass. The first group (All AA) shows the total percentage of non-recovered residue positions of all amino acid types. The second group (100–130 Da) shows the percentages of non-recovered residue positions of amino acid types with a mass between 100 Da and 130 Da, and the third group shows the percentages of non-recovered residue positions of amino acid types with a mass over 130 Da.

To better understand the sequence recovery results, we analyzed the percentage and type of residue positions that were **not** recovered by each method. Over all the designed sequences, rigid DEE failed to recover 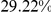 of the designed native residues, while iMinDEE failed to recover 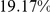, a 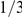 reduction in non-recovered residues. This improvement is highly significant, but the results are more illustrative if we specifically analyze the recovery of large residues and residues with more than one flexible dihedral. If we consider all 13 amino acids with more than one flexible dihedral, rigid DEE failed to recover 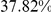 while iMinDEE failed to recover 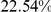 of native residues, a 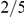 reduction in non-recovered amino acid positions (Figure 10A). If the bulkiest residues (those with a mass over 130 Da: Trp, Phe, Tyr, Arg, Met, and His) are considered, rigid DEE failed to recover 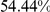 while iMinDEE only failed to recover 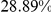, a one-half reduction in non-recovered residues. (Figure 10B). In Table S1 we show a summary of recovered residue positions classified by each amino acid type.

## Discussion

We show here and in previous work [9], [18], [19] that rotamers pruned by rigid DEE can often minimize below the rigid GMEC. Specifically, in 68 of our test systems (Figure 3), MinDEE finds different rotamers for the minGMEC than for the rigid GMEC, as well as different amino acid sequences (in some cases differing in over half of the amino acids) in 66 of the designed protein cores. This demonstrates the importance of using continuous rotamers to find the true minimum energy conformation given the input energy function. In addition, we have developed a new algorithm, iMinDEE, which greatly increases the efficiency of searching over continuous rotamers during protein design.

Stable wild-type proteins have well-packed cores, and mutations that decrease core packing can result in unstable or misfolded proteins [20]–[22]. This is important for our designs because all of the residues that we selected are part of the protein core and have low solvent accessibility (see *Materials and Methods*). In nearly all of our designs the mutated side chains of the minGMEC have a larger volume than those of the rigid GMEC (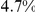 on average, as high as 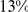). In an average example, 1ZZK with 12 redesigned amino acids and a volume difference of 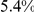, the rigid GMEC and the minGMEC differ in four amino acids: three residues are larger in the minGMEC (M20, M47, I70 in the minGMEC vs. V20, T47, T70 in the rigid GMEC ), and just one residue is smaller (A73 in the minGMEC vs. S73 in the rigid GMEC). Rigid DEE selects a sequence with much smaller amino acid side chains because it cannot find a low energy conformation for the minGMEC sequence. Since overpacking of the minGMEC is unlikely because all of the minGMEC conformations have good vdW potential energies, this increase in volume supports better packing of the minGMEC with respect to the rigid GMEC. Therefore, we believe that modeling continuous rotamers in protein design will reduce the misfolding and increase the stability of predicted proteins.

To further evaluate the biological relevance of our results we performed native sequence recovery with rigid DEE and iMinDEE. iMinDEE obtained significant improvements over rigid DEE in sequence recovery. This shows the importance of fully exploring the protein structural landscape to find the lowest energy structures according to the energy function. Previously, sequence recovery has been used to demonstrate the importance of incorporating desolvation penalties into a protein design energy function [23]. These penalties are usually considered essential for protein design because they account for the hydrophobic effect, which drives protein folding [24]. Interestingly, our results show that the increase in sequence recovery obtained using continuous rotamers is comparable to the increase in sequence recovery obtained by incorporating implicit solvation [23]. This implies that accurately modeling continuous rotamers is as vital to computing accurate designs as incorporating sophisticated energy terms.

It is informative to categorize our sequence recovery results by amino acid mass: (i) *small-mass* amino acids with a mass less than 100 Da (Val, Ala, Gly, and Ser); (ii) *medium-mass* amino acids, with a mass between 100 Da and 130 Da (Asp, Lys, Ile, Gln, Asn, Leu, Glu, Thr); and *large-mass* amino acids, with a mass over 130 Da (Trp, Phe, Tyr, Arg, Met, His). Our results show that, in a rigid-rotamer model, the large-mass residues are recovered significantly less frequently than the small-mass residues. We show that rigid DEE recovered 83.55% of the small-mass residues, but only 45.56% of the large-mass residues. By using a continuous-rotamer model the difference in native sequence recovery of the large-mass residues vs. the small-mass residues is much smaller. iMinDEE recovered 86.54% of the small-mass amino acids and 71.11% of the large-mass amino acids. This further demonstrates that continuous rotamers are necessary to model large amino acids because they are sensitive to small changes in 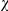 angles.

One might think that increasing the size and resolution of the rotamer library would allow rigid DEE to find the minGMEC. Although this is true in the limit, it is impractical to systematically increase the size of the rotamer library because the side chains of amino acids have many degrees of freedom. If flexibility is handled through more sampling, the protein designer must determine on an *ad hoc* basis what additional sampling should be done within the limits of computational feasibility to allow an angle to deviate from ideal rotamer values. We show in this work that increasing the rotamer library by diversifying the 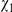, or 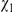 and 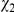 dihedrals still fails to find sequences identical to the minGMEC, and in many cases causes the search to become intractable.

With the introduction of iMinDEE we show that continuous rotamers can efficiently be searched to find the minGMEC. Our pruning results (Figure 7) show that iMinDEE always prunes significantly more rotamers than MinDEE. This increase in pruning greatly reduces the number of protein conformations that A* must search through to find the minGMEC. Remarkably, iMinDEE often prunes close to as many rotamers as rigid DEE. The comparison between iMinDEE/MinDEE and rigid DEE pruning is somewhat complex to interpret since rigid DEE pruning is often incorrect relative to the MinDEE criterion, and the minGMEC is in most cases pruned by rigid DEE. It could also be argued that MinDEE intrinsically should not prune as much as rigid DEE, because its correctness criterion is more stringent (i.e. minimization-aware). Nevertheless, we show that the pruning of MinDEE can be greatly increased while still maintaining correctness. Both MinDEE and iMinDEE have identical outputs, and both guarantee not to prune the minGMEC, and yet iMinDEE prunes orders of magnitude more conformations in all cases.

Pruning with iMinDEE for each design system is greatly affected by the 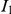 value for that system. The results in Figure 8 show that the performance of iMinDEE can be improved by reducing the value of 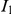. 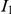 is defined as the difference between 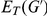 and 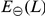 (Eq. (8)). Hence, 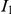 can potentially be reduced either by finding a conformation 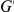 with a lower energy, or by improving the lower bound on the energy of 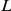 (see Figure 6). First, to find a low-energy conformation for 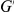, the 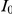 parameter of the iMinDEE algorithm must be chosen with care. While a large 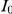 can lead to very little pruning during the first iMinDEE pruning step, a very small 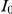 could prevent a low-energy minimized conformation (i.e. a low energy conformation 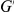, see Figure 6) from being found. This would cause 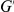 to have a high energy and make 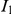 needlessly large. Second, to improve the lower bound on the energy of 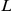 requires improving all of the rotamer energy bounds. The example in Figure 9 shows a case where a poor lower bound on the energy of 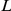 can arise because iMinDEE decomposes the system into rotamer pairs and uses bounds on these pairs to compute the total lower energy bound. One way to prevent this would be to compute lower bounds in a four-wise manner (Arg44 would compute the lower bound with all combinations of neighbors), but this would increase the complexity of the problem by forcing 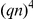 bounds computations (where 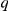 is the number of rotamers per residue, and 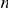 the number of mutable residues). If a four-wise bounds computation solved this specific case, there might be other cases where a higher-order, 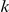-wise computation might be necessary. However, 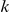 is most likely effectively bounded by a small constant. Improving these bounds as well as choosing an optimal 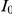 for each design system represents an interesting future research direction.

Our results suggest that the optimal value of 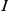 (Eq. (6)) measures the difficulty of accurately designing a given protein system for any pairwise-energy based design algorithm. First, we observed that larger 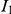 values resulted in less iMinDEE pruning (Figure 8). We also found that rigid DEE with RL2 fails to complete the design search for proteins where iMinDEE computed a large 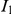 value. These results suggest that large 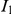-value systems represent difficult design problems for any pairwise-energy based design algorithm. However, since the value computed for 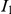 is dependent on the value of 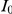 chosen in the iMinDEE algorithm (as described above), it is likely that the optimal value of 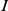, which is approximated by 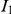, reflects the intrinsic difficulty of a design problem. Therefore, we believe that 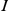, which can be approximated by 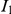, measures an intrinsic degree of difficulty of any design run.

Our previous work, the Backbone DEE (BD) [18] and Backrub DEE (BRDEE) [19] algorithms, showed that we can provably incorporate backbone flexibility into protein design, similar to how MinDEE incorporates side-chain flexibility. Therefore, we can expect an analysis of continuous versus rigid backbone flexibility to yield similar results to those presented here, and that the iMinDEE algorithm presented here can be extended to improve the pruning efficiency of the BD and BRDEE algorithms.

### Relevance for non-DEE/A*-based protein design methods

In this work we show that incorporating continuous rotamers into protein design algorithms can lead to substantially improved design predictions. We used the DEE/A* framework to demonstrate these gains, but our results are applicable to any design method that uses a similar protein design model. As defined in the *Introduction*, the protein design model defines both the input to the algorithm (i.e. energy function and rotamer library) and how the redesigned protein can move (i.e. rigid rotamers or continuous rotamers). Imagine we use the same protein design model, but use different algorithms. Because rigid DEE/A* is guaranteed to find the best sequence according to the protein design model, any design method that uses rigid rotamers, such as Faster [25], Monte Carlo [26], or simulated annealing [27], will never find a lower energy sequence than the rigid GMEC found by DEE/A*. Therefore, the energies of the conformations computed by DEE/A* will always be as low or lower than those computed by non-DEE/A*-based methods using the same protein design model. Hence, our DEE-based results provide a bound on the performance of the other methods. Similarly, the iMinDEE/A* algorithms provide a bound on how well any algorithm based on *continuous rotamers* can perform. By using these bounds, we can precisely measure the consequences of using rigid rotamers to approximate continuous rotamers, and obtain general results that are applicable to all other algorithms using either rigid or continuous rotamers. We can therefore guarantee that the limitations of rigid rotamers are as important for other protein design methods as they are for rigid DEE/A*.

The main consequence of using rigid rotamers in the design search is that the search for side-chain conformations that result in low energy protein structures will not be accurate. Our results show that improving the accuracy and realism of the modeled protein flexibility can greatly improve the results of the design search. In our work we used a simple energy function in which every term can be related to physical phenomena, and found that by switching from rigid to continuous rotamers we could discover lower energy sequences and observe large gains in sequence recovery. This demonstrates that if all sequences and structures are not adequately searched to find the lowest energy ones, the most biologically-relevant results are missed. Unfortunately, the importance of accurately searching for the true lowest energy structure and sequence is sometimes overlooked and the inaccuracies are attributed instead to the energy function. Protein design energy functions are constantly improved through careful crafting to better correlate designs with retrospective biological results. Many improvements to energy functions are made through the introduction of complex statistical terms based on structural bioinformatics data and other additional parameters[28], [29]. If the rigid-rotamer search inaccuracies are wrongly attributed to imperfections in the energy function, the results will be used to incorrectly modify the energy function. Therefore, to avoid over-fitting the energy function, accurate flexibility, such as continuous rotamers, should be used during the design process.

It is often assumed in the protein design field that even if the minGMEC and the rigid GMEC are different, minimizing and reranking the top 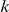 results from a rigid approach can lead to finding the minGMEC [30]. Several of our results suggest that this is very likely to not be the case, and the minGMEC would never even be considered by any rigid-rotamer method. First, the enormous difference in sequence and amino acid composition between the rigid GMEC and the minGMEC is striking: in some cases the difference is over 60%. Second, the side chains of the amino acids in the rigid GMEC tend to have a smaller volume than the side chains of the minGMEC, suggesting that unavoidable clashes in a rigid-rotamer model would make the rotamers of the minGMEC unable to sterically fit in a rigid-rotamer environment. We analyzed the conformations of the minGMEC in all of our 69 designs and found that if the continuous rotamers were replaced by their closest (i.e. in 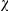-angle space) rigid-rotamer counterpart at each position, most of the designs would obtain high-energy steric clashes (up to 1000 kcal/mol higher than the rigid GMEC). Even when the rigid-rotamer library was expanded, the new library could not capture the low-energy sequences of the continuous rotamers. Thus, contrary to conventional wisdom, rigid rotamers are always a severely limited approximation to continuous rotamers.

Any protein design algorithm that switches from using rigid rotamers to continuous rotamers will expand the side-chain search space it explores. As the sequence and conformation space increases, it is always desirable to quickly and efficiently reduce the space to make the search more tractable. In this work we presented the novel iMinDEE pruning condition which can reduce the conformational space by many orders of magnitude. After iMinDEE pruning we search the remaining conformational space with the A* search algorithm. We use A* as the search algorithm because it is guaranteed to find the optimal answer, but any search algorithm can be used in combination with iMinDEE. In fact, an approach analogous to using iMinDEE with a different continuous-rotamer search algorithm is frequently used in rigid-rotamer protein design protocols. Rigid DEE was used as a filter for Monte Carlo searches [31] or for the FASTER algorithm [25]. iMinDEE can therefore have considerable impact for any protein design algorithm that uses continuous rotamers.

## Materials and Methods

### Protein test sets

Crystal structures of protein chains with a maximum percentage sequence identity of 10% and a maximum resolution of 1.3 Å were chosen using the PISCES protein culling server [32]. In addition, the protein chains were restricted to have a maximum length of 100 residues. The protein crystal structures were gathered from the PDB and further curated by adding hydrogens [33] and removing waters and ions. Residues with missing side chains were either removed entirely or the missing atoms were added using the King software package [34]. In total, 69 protein structures were selected for the test set.

### Design runs

For each protein in the test set, a redesign to find low energy sequences for the initial backbone (a mutation search) was conducted. Each mutation search was designed so that approximately 12–15 core residues of the protein would be mutable. Core residues were chosen by finding all residues with a side-chain relative solvent accessible surface area (SASA) less than either 5%, 10%, or 20%. SASA values were determined with the program NACCESS [35]. If a protein had less than 12 residues with 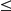
20% SASA, only these residues were allowed to mutate. Each mutable residue was allowed to take on its wild-type identity and several other amino acid types. The mutant amino acid types were determined by finding the 5–7 most likely amino acid type substitutions based on the BLOSUM62 matrix [36]. The AMBER [37] energy function and the Richardson's Penultimate Rotamer Library [38] were used as input to the algorithm. Each design run consisted of three steps: (1) A pairwise energy matrix precomputation between all pairs of side chains [9], and a minimum energy bound matrix precomputation for MinDEE [9] and iMinDEE; (2) Several rounds of DEE/MinDEE/iMinDEE pruning to reduce the search space; and (3) An A* conformational search [9], [13] of the remaining space. Each design was run in an Intel Xeon machine with at least 4 GB of dedicated RAM and at least 2.50 Ghz of processor speed.

### DEE pruning

The protein design runs were done using rigid DEE, MinDEE, and iMinDEE. All three algorithms performed an initial steric filter to prune rotamers that could not minimize away from a clash with the template. Implementations of Goldstein DEE [39], Goldstein Pairs, and Split Flags [40] were used for all three algorithms, while Bounds Pruning [9], [41] was used for rigid DEE and MinBounds Pruning for MinDEE and iMinDEE [9]. iMinDEE was run with an initial interval value 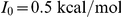 for all the mutation searches. 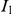 was chosen based on the minimum difference between the lowest-energy bound and the lowest minimized energy found in the first run.

### Energy function

To evaluate molecular energetics we used an energy function very similar to the energy function used for our previously described, empirically successful protein designs [2]–[4]. The energy function is composed of the following energy terms: (1) attractive-repulsive van der Waals forces, and coulombic electrostatics with a distance-dependent dielectric from the AMBER energy function [37]; (2) implicit solvation terms from the Lazardis Karplus EEF1 solvation model to account for the hydrophobic effect [24]; and (3) entropic penalties [10], [42] and reference energies [15] to account for entropy and energetics of the unfolded protein state. The total energy for a protein structure was calculated by computing a linear combination of all the energy terms, using weightings for the terms as described below.

The weighting of each energy term is important for accurate results and most successful protein designs perform some training of the energy parameters [3], [16], [29]. We trained our energy function by performing protein core redesigns on 9 structures from the PDB database that were not in the set of 69 structures used in this study. The structures for the training set (PDB ids: 1fus, 1ifc, 1lkk, 1plc, 1poa, 1rro, 1whi, 2rhe, and 2trx) were selected from the Richardson's Top 100 database of high-quality curated protein structures [43]. All of them were reprotonated according to the PDB v3 [33] standard and energy minimized with Sander [37]. Residues with less than 20% SASA were selected to mutate; the low-SASA residues were split into groups of 10–15 highly-interacting residues each.

Training was performed by redesigning each group of low-SASA residues with rigid DEE/A* and allowing each amino acid to be mutated to the same 5–7 amino acids allowed in the design runs, which were based on the BLOSUM62 matrix [36]. In addition, each wild-type rotamer was added to the rotamer library. Each redesign was first run using 21 different coarse parameter combinations of solvation and dielectric constant defined by a 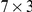 grid with solvation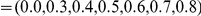 and dielectric constant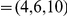. The optimal value found was solvation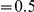 and dielectric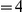. We then set solvation to 0.5 and dielectric to 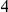 and performed a local minimization by scaling atom radii. Scaling down the radii of atoms decreases the effect of the repulsive term in the van der Waals energy term. We used scales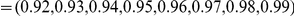. The optimal atom radii scaling factor was determined to be 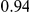.

### Native sequence recovery

Each of the 69 protein systems used in our runs was manually analyzed for ligands or co-factors that appeared close to core-residues. Structures with ligands or co-factors in close contact to the mutable design residues were not considered, because functional residues tend to be optimized for functionality and not to stabilize the monomeric structure [17]. 43 protein structures remained after removing those with interacting ligands or co-factors. Each mutation search was set up so that approximately 12–15 core residues of the protein would be mutable. Core residues were chosen by finding all residues with a side-chain relative solvent accessible surface area (SASA) less than either 5%, 10%, or 20%. SASA values were determined with the program NACCESS [35]. If a protein had less than 12 residues with 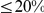 SASA, only these residues were allowed to mutate. Each mutable residue was allowed to take on its wild-type identity and 5–7 other amino acid types. The mutant amino acid types were determined by finding the 5–7 most likely amino acid type substitutions based on the BLOSUM62 matrix [36]. The native rotamers were not included in the native sequence recovery experiments. Native sequence recovery was then performed on the 43 proteins with PDB ids: 1lni, 1ok0, 1psr, 1t8k, 1u2h, 1usm, 1wxc, 1zzk, 2cov, 2fhz, 2hs1, 2r2z, 3d3b, 3dnj, 1l9l, 1r6j, 1u07, 1ucs, 1vbw, 1y6x, 2hin, 2j8b, 2p5k, 2wj5, 3g21, 3hfo, 3jtz, 1aho, 1f94, 1oai, 1vfy, 2b97, 2cc6, 2cg7, 2dsx, 2fma, 2gom, 2hba, 2hlr, 2ic6, 3g36, 3i2z, and 1i27.

## Supporting Information

Table S1Summary of native residue positions recovered by each method categorized by amino acid type. 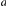 Total number of wild-type instances of each amino acid type in the native sequences of the redesigned proteins. 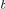 Total number of residue positions recovered in the rigid GMEC computed by rigid DEE. 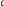 Percentage of residue positions recovered in the rigid GMEC computed by rigid DEE. 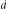 Total number of residue positions recovered in the minGMEC computed by iMinDEE. 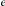 Percentage of residue positions recovered in the minGMEC computed by iMinDEE.(PDF)

Text S1 Proof of Proposition 1.
(PDF)
