# Supplementary material for: Protein Design Using Continuous Rotamers
Source: PLoS Comput Biol. 2012 Jan 12;8(1):e1002335. doi: 10.1371/journal.pcbi.1002335 (PMC3257257; doi:10.1371/journal.pcbi.1002335)
Supplement: Table S1 — Summary of native residue positions recovered by each method categorized by amino acid type. Total number of wild-type instances of each amino acid type in the native sequences of the redesigned proteins. Total number of residue positions recovered in the rigid GMEC computed by rigid DEE. Percentage of residue positions recovered in the rigid GMEC computed by rigid DEE. Total number of residue positions recovered in the minGMEC computed by iMinDEE. Percentage of residue positions recovered in the minGMEC computed by iMinDEE. (PDF) [file pcbi.1002335.s001.pdf]

Supplementary Table S1

Amino acid type	Wild-type count[a]	rigidGMEC recovery[b]	rigidGMEC recovery[c]	minGMEC recovery[d]	minGMEC recovery[e]
ASP	7	6	85.71%	7	100.00%
LYS	2	2	100.00%	1	50.00%
ILE	77	52	67.53%	59	76.62%
TRP	12	6	50.00%	11	91.67%
GLY	34	33	97.06%	34	100.00%
PHE	37	15	40.54%	25	67.57%
GLN	4	3	75.00%	3	75.00%
SER	19	7	36.84%	13	68.42%
ASN	13	10	76.92%	9	69.23%
LEU	81	57	70.37%	69	85.19%
VAL	110	93	84.55%	91	82.73%
TYR	17	4	23.53%	8	47.06%
GLU	1	0	0.00%	1	100.00%
ARG	3	1	33.33%	2	66.67%
THR	21	9	42.86%	13	61.90%
ALA	68	60	88.24%	62	91.18%
MET	17	12	70.59%	14	82.35%
HIS	4	3	75.00%	4	100.00%
Total	527	373	70.78%	426	80.83%
