# Supplementary material for: Protein Design Using Continuous Rotamers
Source: PLoS Comput Biol. 2012 Jan 12;8(1):e1002335. doi: 10.1371/journal.pcbi.1002335 (PMC3257257; doi:10.1371/journal.pcbi.1002335)
Supplement: Text S1 — Proof of Proposition 1. (PDF) [file pcbi.1002335.s002.pdf]

Supplementary Text S1

In this document we present a proof of Proposition 1. We restate both the iMinDEE criteria, and Proposition 1 for clarity.

The iMinDEE criterion is:

$$E_{\ominus}(i_r) + \sum_{j \neq i} \min_s E_{\ominus}(i_r, j_s) > E_{\ominus}(i_t) + \sum_{j \neq i} \max_s E_{\ominus}(i_t, j_s) + I. \tag{7}$$

Proposition 1. *When Eq. (7) holds, rotamer i_r can be provably pruned from the search space because it cannot be part of the minimized global minimum energy conformation (minGMEC).*

Proof. Let G be the rotamer vector that minimizes into the minimized-GMEC and $E_T(G)$ be the energy of the minimized-GMEC. Let $A = G_{i_g \to i_t}$ be the rotamer vector G where rotamer i_g is replaced with i_t. Let $E_{\odot}(i_r|A)$ be the internal energy of i_r when rotamer vector A is minimized and let $E_{\odot}(i_r, j_s|A)$ be the pairwise energy of i_r and j_s when A is minimized. Also, let L be the rotamer vector with the lowest minimum bound. Note, L and G are most likely not the same rotamer vector. By definition we know that

$$E_{\ominus}(A) \geq E_{\ominus}(L).$$

Adding $E_T(G)$ to both sides gives:

$$E_{\ominus}(A) + E_T(G) \geq E_T(G) + E_{\ominus}(L).$$

Moving $E_{\ominus}(L)$ to the left side and using the definition, $I \geq E_T(G) - E_{\ominus}(L)$:

$$E_{\ominus}(A) + I \geq E_T(G).$$

Expanding $E_{\ominus}(A)$ and $E_T(G)$:

$$\begin{aligned} E_{\ominus}(i_t) + \sum_{j \neq i} E_{\ominus}(i_t, j_g) + \sum_{j \neq i} E_{\ominus}(j_g) + \sum_{j \neq i} \sum_{k \neq i} E_{\ominus}(j_g, k_g) + I \\ \geq E_{\odot}(i_g|G) + \sum_{j \neq i} E_{\odot}(i_g, j_g|G) \\ + \sum_{j \neq i} E_{\odot}(j_g|G) + \sum_{j \neq i} \sum_{k \neq i} E_{\odot}(j_g, k_g|G). \end{aligned} \tag{8}$$

We can use the fact that

$$\sum_{j \neq i} E_{\odot}(j_g|G) \geq \sum_{j \neq i} E_{\ominus}(j_g),$$

$$\sum_{j \neq i} \sum_{k \neq i} E_{\odot}(j_g, k_g|G) \geq \sum_{j \neq i} \sum_{k \neq i} E_{\ominus}(j_g, k_g)$$

and substitute these two equations into Eq. (8) which simplifies to:

$$E_{\ominus}(i_t) + \sum_{j \neq i} E_{\ominus}(i_t, j_g) + I \geq E_{\odot}(i_g|G) + \sum_{j \neq i} E_{\odot}(i_g, j_g|G).$$

We can further relax this inequality by using the fact that $E_{\odot}(i_g|G) \geq E_{\ominus}(i_g)$ and $\sum_{j \neq i} E_{\odot}(i_g, j_g|G) \geq \sum_{j \neq i} \min_s E_{\ominus}(i_g, j_s)$ and substitute into the above inequality:

$$E_{\ominus}(i_t) + \sum_{j \neq i} E_{\ominus}(i_t, j_g) + I \geq E_{\ominus}(i_g) + \sum_{j \neq i} \min_s E_{\ominus}(i_g, j_s).$$

Since we will not know G during the computational search, and since $\sum_{j \neq i} \max_s E_{\ominus}(i_t, j_s) \geq \sum_{j \neq i} E_{\ominus}(i_t, j_g)$, we again relax the inequality:

$$E_{\ominus}(i_t) + \sum_{j \neq i} \max_s E_{\ominus}(i_t, j_s) + I \geq E_{\ominus}(i_g) + \sum_{j \neq i} \min_s E_{\ominus}(i_g, j_s). \tag{9}$$

Now, if there exists a rotamer i_r that meets the criterion of Eq. (7) we can substitute the left hand terms of Eq. (7) into Eq. (9):

$$E_{\ominus}(i_r) + \sum_{j \neq i} \min_s E_{\ominus}(i_r, j_s) > E_{\ominus}(i_g) + \sum_{j \neq i} \min_s E_{\ominus}(i_g, j_s).$$

Thus, if the pruning condition holds, rotamer i_r cannot be i_g, so i_r can be pruned from the rotamer search. $\square$
